# Worcester Infirmary

**Published:** 1830-11

**Authors:** 


### WORCESTER INFIRMARY.

### *Purulent Ophthalmia, treated with Argent. Nitratum.* By Mr. Herbert Cole.*

April 15, 1830. John Griffiths, a fine child, six weeks of age, was brought here with purulent ophthalmia of both eyes, the cornea of left eye quite opaque from the ulcers which covered it; the right eye but little better. The eyes have been affected ever since the child's birth. The mother had leucorrhœa at the time it was born. The child is now out of health, being much purged. Its stools green, sour, and slimy. ℞. Argenti Nitratis, gr. x.; Aq. distill. f. ℥i. m. f. gttæ. oculis b. d. instillandæ. Sumt. 6ta qq. hora Hydrarg. c. Creta gr. vi. Hydrarg. Submur. gr. $\frac{1}{4}$ partem. Emplast. lyttæ nuchæ applicetur.

21st. Child's health is somewhat improved. The stools are of a better colour, and not so frequent; discharge not so profuse from the eyes, which are less inflamed. Contin. guttæ; omitt. pulveres. Cap. Solut. Magnes. Sulph. f. ℥ss. pro re nata.

25th. Eyes very much improved; left eye, which was the worst, is now nearly well. The child gets flesh.

26th. Repet. emplast. lyttæ nuchæ.

In about a month after its admission, the child was discharged, cured; the corneæ of both eyes being quite transparent, and the sense of vision quite restored.

* Midland Med. and Surg. Reporter.

